# Proteinaceous Toxins in the Mucus and Proboscis of the Ribbon Worm *Cephalothrix* cf. *simula* (Palaeonemertea: Nemertea)

**DOI:** 10.3390/toxins18010017

**Published:** 2025-12-27

**Authors:** Vasiliy G. Kuznetsov, Daria I. Melnikova, Sergey V. Shabelnikov, Timur Yu. Magarlamov

**Affiliations:** 1 A.V. Zhirmunsky National Scientific Center of Marine Biology, Far Eastern Branch, Russian Academy of Sciences, 690041 Vladivostok, Russia; 2 Institute of Cytology, Russian Academy of Sciences, 194064 St. Petersburg, Russia

**Keywords:** nemertean toxins, proteomics, ribbon worm, HPLC–MS/MS, cysteine-rich peptides, pseudocnidae, mucus, proboscis, RT-qPCR, *Cephalothrix* cf. *simula*

## Abstract

*Cephalothrix* cf. *simula* is a highly toxic ribbon worm of the class Palaeonemertea, known for its high concentrations of tetrodotoxin. Recent transcriptomic and proteomic studies across Nemertea have revealed that species from all classes possess a diverse array of protein and peptide toxins, which are associated with unicellular glands of the proboscis and the integument epithelium. Previous studies have identified a large number of putative toxins in the transcriptome of *C*. cf. *simula*; however, corresponding proteomic data have so far been lacking. This study presents the first comprehensive analysis of the mucus and proboscis proteome of *C*. cf. *simula* using high-performance liquid chromatography–tandem mass spectrometry. We identified three putative toxins in the proboscis and three in the mucus. Additionally, four cysteine-rich peptides with putative toxic activity were identified in the mucus and one in the proboscis. The expression of the corresponding genes in both tissues was quantified using quantitative real-time PCR. The toxin compositions of the proboscis and mucus showed clear signs of functional specialization, with no overlapping toxins and tissue-specific patterns of gene expression. Feeding experiments combined with transmission electron microscopy confirmed the involvement of specialized proboscis structures, pseudocnidae, in delivering toxins into the prey.

## 1. Introduction

Nemerteans, or ribbon worms, are a phylum of predominantly benthic marine invertebrates whose biology is closely associated with the production of bioactive secretions [1,2]. The phylum Nemertea is currently divided into three classes: Palaeonemertea, Pilidiophora (including Hubrechtiiformes and Heteronemertea), and Hoplonemertea [3,4,5]. Despite their limited locomotor capacity and soft-bodied morphology, most nemerteans are generalist predators with a broad diet encompassing annelids, crustaceans, mollusks, and other nemerteans [2,6,7]. Predation pressure on nemerteans is relatively low, as they are infrequently consumed by typical benthic predators, such as fish and decapod crustaceans [2,8,9,10,11].

To provide effective prey capture and defense, nemerteans rely on a combination of mechanical and chemical adaptations. Mechanical adaptations include an eversible proboscis apparatus used for prey capture and extensive mucus secretions that facilitate environmental interaction and protection [12]. Chemical adaptations involve toxins stored in glandular cells of both the proboscis and the integument epithelium [13,14,15,16,17,18,19]. To stab prey and inject toxins, mono- and polystiliferous hoplonemerteans use a specialized calcified spine, or stylet, located in the middle of the proboscis [13,20,21,22,23]. In contrast, the proboscis of palaeonemerteans and pilidiophorans, except for certain genera, contains rod-shaped secretory granules called pseudocnidae, which house a hollow, thread-like tubule (core) thought to be capable of everting and penetrating the integument of prey [24]. However, to date, there are no confirmed cases of pseudocnidae discharge during prey capture.

Studies of nemertean toxins began in the late 1960s with the discovery of pyridine-derived alkaloids that affect nicotinic acetylcholine receptors, including the well-known anabaseine and nemertelline [25,26]. Pyridine alkaloids have been identified only in Hoplonemertea [26,27,28,29]. Subsequently, tetrodotoxin (TTX), a potent blocker of voltage-gated sodium channels widely known from pufferfish, was reported in several nemertean species [30,31,32]. Although TTX and its analogs occur across all nemertean classes, most evidence comes from palaeonemerteans of the *Cephalothrix simula* s.l. species complex, which inhabit Japanese and Russian waters [33]. The first polypeptide neurotoxins, causing crayfish paralysis, were observed in heteronemerteans of the genus *Lineus* in the 1980s [2]. To date, all physically isolated and biochemically characterized nemertean-derived peptide toxins, including cytolysins A II-IV, neurotoxins B I-IV, α-nemertides, and parborlysins, have been obtained from Pilidiophora [1].

Recent genomic, transcriptomic, and proteo-transcriptomic studies indicate that nemerteans from all classes harbor a diverse array of putative protein and peptide toxins [19,34,35,36,37,38,39,40]. Molecular evolutionary analyses show that nemertean toxin repertoires have been shaped by extensive gene duplication, neofunctionalization, and positive selection, leading on one hand to lineage-specific expansions of toxin families and on the other to the retention of highly conserved structural scaffolds [37,38]. Many toxin families exhibit signatures of convergent evolution, sharing structural motifs, domain architectures, or functional traits with toxins from arthropods, cnidarians, mollusks, and vertebrates [19,34,35,36,38]. In contrast, nemertean-specific inhibitor cystine knot (ICK) peptides and cytolysins underscore the biochemical novelty and pharmacological potential of nemertean secretory systems [39,40]. Nevertheless, a large fraction of putative nemertean toxins remain unannotated and functionally uncharacterized.

This study focuses on the palaeonemertean *Cephalothrix* cf. *simula*, one of the cryptic species within the *C*. *simula* s.l. species complex [41,42,43]. Species in this complex are notable for their high concentrations of TTX [31,44,45,46,47], while other toxins in the group remain poorly studied. In the study by Vlasenko et al. [37], the transcriptome of *C*. cf. *simula* was sequenced, revealing a high number of putative toxin transcripts. These transcripts include enzymes, ion-channel inhibitors, pore-forming toxins, proteinase inhibitors, neurotoxins, hemostasis-impairing toxins, and other toxin candidates, with ion-channel inhibitors representing the most highly expressed group. Here, we performed a comparative proteomic analysis of the mucus and proboscis of *C*. cf. *simula* from the Sea of Japan. We identified candidate protein and peptide toxins, characterized cysteine-rich peptides (CRPs) with putative toxic activity, and quantified the expression of their encoding genes in both tissues. Feeding experiments combined with transmission electron microscopy (TEM) have been conducted to investigate the role of pseudocnidae in toxin injection during prey capture in nemerteans. These findings provide new insights into the functional differentiation and ecological roles of nemertean secretions.

## 2. Results

For proteomic analysis, four proboscis and six mucus samples, each obtained from a distinct *C*. cf. *simula* specimen, were analyzed. Analysis was conducted using the previously published transcriptome of *C*. cf. *simula* [37] as the reference database for protein identification. Analysis was performed in ten independent runs using a matrix-assisted laser desorption/ionization time-of-flight/time-of-flight (MALDI-TOF/TOF) mass spectrometer. In the proboscis, 360 proteins and peptides were identified, most of which were cytoplasmic or membrane-associated, while 50 were predicted to be extracellular or contain a signal peptide. These included one peptide (<100 amino acids) and 49 proteins, 39 of which were identified through the UniProtKB/Swiss-Prot database [48]. According to UniProt annotations, the dataset comprised 12 enzymes, 10 membrane/adhesion proteins, 7 structural proteins, 5 regulatory/signaling proteins, 2 serine-protease inhibitors, 1 histone, 1 ribosomal protein, and 1 centipede-type venom neurotoxin (Table 1; Supplementary Table S1).

A total of 35 proteins and 5 peptides were identified in the mucus of *C*. cf. *simula*, including 23 proteins and 5 peptides predicted to contain signal peptides. Most sequences showed no significant similarity to known sequences. Of the 10 annotated proteins, 2 were signaling, 2 structural, 1 a housekeeping metabolic enzyme, 2 enzymatic toxins, and 3 corresponded to the same pore-forming toxin (Table 2; Supplementary Table S2).

Cysteine-rich peptides from the mucus and proboscis of *C*. cf. *simula* were used for further analysis with ConoServer (https://www.conoserver.org/, accessed on 11 November 2025). Only mature peptides of up to 100 amino acids with a predicted signal sequence and at least four cysteine residues were considered. Four candidate peptides from the mucus (ORF|114503, ORF|035733, ORF|002160, ORF|001398) and one from the proboscis (ORF|020829) were selected. None of the selected peptides had UniProt annotations matching known sequences. Mucus peptides ORF|114503 and ORF|035733 were classified as cysteine framework XXV (C–C–C–C–C–C), ORF|002160 as framework IX (C–C–CC–C–C) (Supplementary Figure S1). The proboscis peptide ORF|020829 and the mucus peptide ORF|001398 did not match any known conotoxin frameworks. Searching for homologous sequences in the transcriptomes of 12 nemertean species analyzed by Vlasenko et al. [37] revealed putative orthologs of the selected peptides in the transcriptome of *Cephalothrix hongkongiensis*. Pairwise alignments using Jalview v2.11.5.1 [49] revealed high amino acid identity (84.4–100%) between the corresponding sequences (Supplementary Figure S1).

The tertiary structures of the selected peptides were predicted using Phyre2 [50] and compared with known peptides to assess structural similarity and potential functional relationships. High-confidence predictions (>80%) were obtained for the mucus peptide ORF|114503 and the proboscis peptide ORF|020829, showing similarity to NEUROTOXIN I (confidence 85.1%; coverage 48%) and MU-AGATOXIN-I (confidence 86.9%; coverage 57%), respectively. The mucus peptide ORF|035733 aligned with an anti-hypertensive, anti-viral protein (confidence 75.6%; coverage 35%), while ORF|001398 and ORF|002160 showed low similarity to the TRPV1 inhibitory peptide Tst2 (confidence 44.3%; coverage 56%) and a metalloproteinase inhibitor protein (confidence 43.9%; coverage 39%), respectively. Predicted structures of ORF|114503 and ORF|020829 were structurally aligned with their closest Protein Data Bank (PDB) templates using UCSF Chimera v1.19 [51]. Visualization and superimposition revealed that both CRPs adopt a conserved disulfide-stabilized core fold similar to their respective templates, while notable divergence was observed in peripheral loop regions and terminal segments (Supplementary Figure S2).

Putative functional classes of selected peptides were also predicted using CSPred v 1.1 [52]. Mucus peptides ORF|035733 and ORF|002160 were classified as antimicrobial with high probability score > 0.8 (Table 3). Mucus peptide ORF|114503 was predicted to act as an ion channel-blocking neurotoxin with a probability of 0.71. Two other peptides could not be classified by CSPred. To further assess the antimicrobial potential of ORF|035733, molecular surface properties were examined in UCSF Chimera. Electrostatic surface analysis revealed extended positively charged regions, consistent with cationic antimicrobial peptides that preferentially interact with negatively charged microbial membranes. Hydrophobic surface mapping identified discrete hydrophobic patches, suggesting an amphipathic architecture suitable for membrane insertion and disruption (Supplementary Figure S3). Structural interpretation of peptides with low-confidence template matches (ORF|001398 and ORF|002160) was limited and therefore not pursued further.

**Figure 1:**
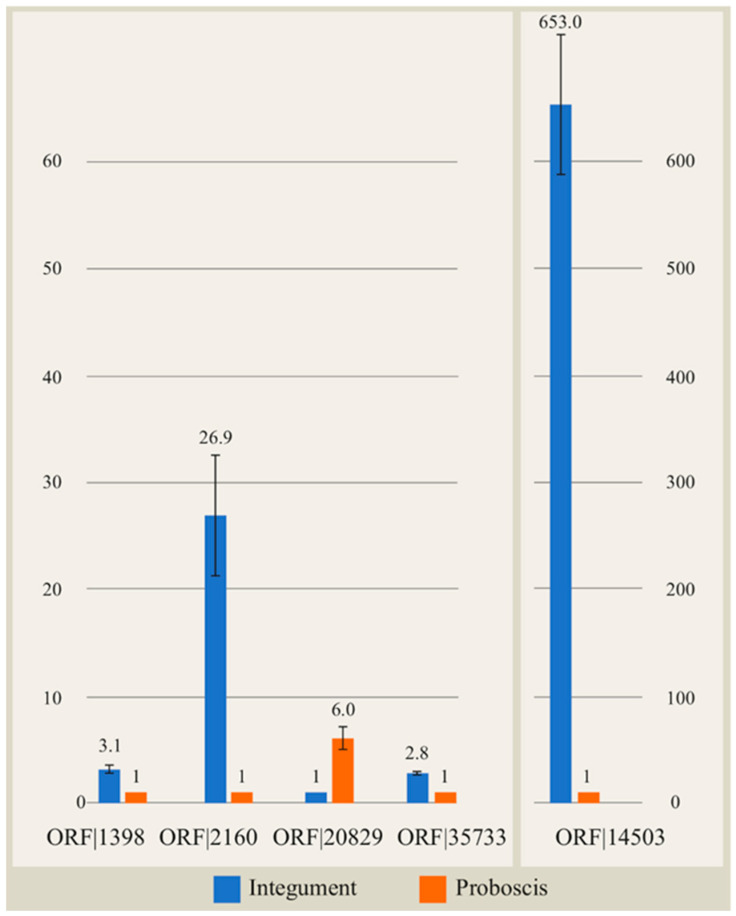
Relative expression levels of selected cysteine-rich peptides identified in the proteome of *Cephalothrix* cf. *simula* in the integument and proboscis. Gene expression levels were quantified by quantitative real-time PCR using the 2^−∆∆Ct^ method. Data represent the mean of three technical replicates ± SEM. Reference gene: *act-1a*. Calibrator sample: proboscis.

The expression levels of genes encoding the selected peptides in the integument and proboscis of *C*. cf. *simula* (Figure 1) were measured by quantitative real-time PCR (qRT-PCR), with actin serving as the reference gene. All mucus peptide genes showed higher expression in the integument than in the proboscis, although the degree of upregulation varied. The ORF|01398 and ORF|035733 genes were expressed approximately 3-fold higher in the integument, whereas ORF|002160 and ORF|114503 showed markedly stronger expression—by 27- and 652-fold, respectively. In contrast, ORF|020829 was expressed 6-fold higher in the proboscis.

**Figure 2:**
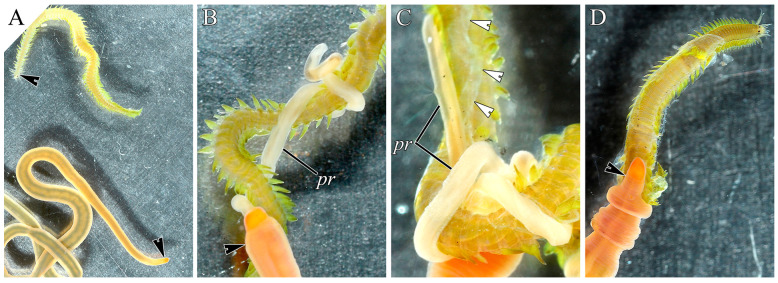
Light microscopy images of *Cephalothrix* cf. *simula* preying on the polychaete *Eulalia* sp. Black arrowheads indicate the heads of the animals. (**A**)—A polychaete crawling near a nemertean. (**B**)—The nemertean attacks the polychaete with its proboscis. (**C**)—The proboscis wraps around the polychaete, forming coils. White arrowheads indicate the mucous strand left by the everted proboscis on the prey’s surface. (**D**)—The nemertean consumes the immobilized polychaete. Abbreviation: pr, proboscis.

The in vivo feeding behavior of *C*. cf. *simula* on polychaetes was examined at the light-optical level by Malykin et al. [53]. In the present study, after detecting the prey (Figure 2A), the nemertean everted its proboscis, which moved along the prey’s body and then wrapped around it, forming coils (Figure 2B,C). The proboscis left a mucous trail on the surface of the polychaete (Figure 2C), which remained on the prey until it was completely consumed. After the attack, the nemertean retracted its proboscis and crawled onto the partially or fully immobilized prey, consuming it (Figure 2D).

**Figure 3:**
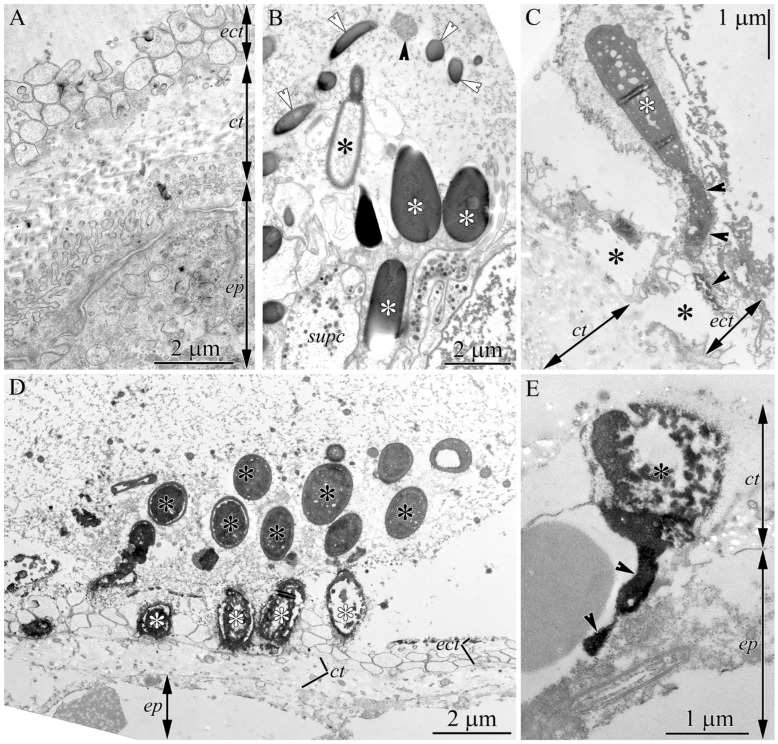
Transmission electron micrographs of *Cephalothrix* cf. *simula* and *Eylalia* sp. (**A**)—*Eulalia* sp., panoramic view of the apical region of the integument of an intact specimen. (**B**)—*C*. cf. *simula*, apical region of the proboscis glandular epithelium showing extruded (black asterisk) and intact (white asterisks) pseudocnidae. Secretory granules of type II granular cells are released (white arrowheads) and partially degraded (black arrowhead). (**C**)—Outer surface of the attacked polychaete associated with discharged pseudocnida (white asterisk). Arrowheads indicate an extruded core of a pseudocnida embedded in the epicuticle of the polychaete. Black asterisks indicate zones of epicuticle lysis. (**D**)—Remnants of nemertean proboscis glandular epithelium on polychaete integument. Pseudocnidae are embedded within the mass (black asterisks) or on its surface (white asterisks). (**E**)—A single discharged pseudocnida (asterisk) in the cuticle of the attacked polychaete. Arrowheads indicate extruded core inside the epithelial cells of the polychaete. Abbreviations: ect, epicuticle; ct, cuticle; ep, epidermis; supc, supportive cell.

Using TEM, we refined the light-optical observations. The integument of intact polychaetes consisted of a single-layered epidermis covered by a cuticle (2.5–3.8 µm thick) and an epicuticle (approximately 2 µm thick) (Figure 3A). In nemerteans, the apical surface of the proboscis glandular epithelium bore papillae of glandular cells and monolayered clusters of mature pseudocnidae, which were surrounded by microvilli of supportive cells (Figure 3B). Some pseudocnidae on the apical surface of the proboscis epithelium had extruded (discharged) cores (Figure 3B, black asterisk). At the site of proboscis contact on the surface of the polychaete, remnants of the proboscis glandular epithelium formed a fibrillar-granular mass in which discharged pseudocnidae were embedded (Figure 3D). Some of these pseudocnidae were within the mass, while others were on its surface facing the polychaete. The mucous strand was in close contact with the polychaete’s integument; at the contact site, peripherally located pseudocnidae either rested on the epicuticle surface (Figure 3C) or were embedded within it (Figure 3D). Some pseudocnidae were located outside the mucus strands and were found within the cuticular layer, while their extruded cores penetrated the epithelial cells (Figure 3E).

## 3. Discussion

Here, we report for the first time the proteomic sequencing of the mucus and proboscis of *C*. cf. *simula*, integrated with previously obtained transcriptomic data [37]. Although 82 putative toxin transcripts were identified in the transcriptome of the nemertean, the proteomic analysis confirmed a total of three putative toxins in the proboscis and three in the mucus (Table 1 and Table 2). However, a substantial portion of secreting proteins—22% from the proboscis and 64% from the mucus—were not annotated in the UniProtKB/Swiss-Prot database.

The only study to date reporting the proteome of a nemertean proboscis was performed on the hoplonemertean *Antarctonemertes valida* [19]. The authors identified 26 putative proboscis-specific toxins, nine of which could be annotated. These included proteins with insulin-like growth factor-binding domains, galactose-binding domains, antistasin-like domains, and pulmonary surfactant–associated protein-like domains. While similar proteins are found in other animal venoms, their specific functions are not clear. In the proboscis of *C*. cf. *simula*, putative toxins included protease inhibitors resembling antistasin and leukocyte elastase inhibitor, and a U-scoloptoxin(05)-Er3a-like neurotoxin. Besides the proboscis of *A*. *valida* [19], antistasin/Kazal-type inhibitors have previously been reported in the mucus of the heteronemerteans *Lineus sanguineus* and *Kulikovia alborostrata* [38,40]. Although leukocyte elastase inhibitors (SERPIN family) have not been detected in nemerteans, other serine protease inhibitors are present in transcriptomes across all classes, with Kunitz-type inhibitors being the most common [37,40]. Snake venom-like serine proteases have also been detected in the mucus of the hoplonemertean *Amphiporus lactifloreus* [36]. Scoloptoxin-like proteins are widespread in nemertean transcriptomes [36,37,38,40] and have been detected in the mucus of *A*. *valida* [19] and in the body of the hoplonemertean *Nemertopsis pamelaroeae* [38]. In the present study, we identified a CRP with elevated expression in the proboscis, whose 3D structure resembles that of the neurotoxin MU-AGATOXIN-I. This structural similarity suggests that the peptide may share mechanistic features typical of agatoxin-like neurotoxins, including interactions with ion channels. The conserved disulfide-stabilized scaffold likely ensures structural stability, whereas variation in peripheral loop regions may modulate target specificity. Similar channel-blocking toxins have been found in the mucus of *L*. *sanguineus* [38] and in the transcriptome of *K*. *alborostrata* [40]. However, sequence homology searches revealed putative orthologs only in the closely related species *C*. *hongkongiensis*.

The proboscis of nemerteans is a long muscular tube formed by an invagination of the anterior end of the body [12,21,54]. When the proboscis is everted, the glandular epithelium occupies an external position and directly interacts with the prey. The glandular epithelium of the proboscis of *C*. cf. *simula* comprises four types of glandular cells [18]. Type III cells contain pseudocnidae, while Types I and II (bacillary) and Type IV (mucoid) cells possess round or oval shaped secretory granules [18]. Malykin et al. [18] showed that Type II bacillary glandular cells are TTX-positive and that the mucus accumulating in the proboscis lumen contains TTX. According to the hypothesis proposed by Montalvo et al. [15], the mucous cell system and its secreted components create a sticky adhesive environment during proboscis eversion that helps retain pseudocnidae on the prey’s skin, thereby increasing tissue damage and facilitating toxin penetration. Malykin et al. [18] proposed that the pseudocnidae-containing cells of *C*. cf. *simula* discharge their cores in response to mechanical stimulation while remaining situated in the apical region of the bacillary cells. Our observations showed that during attacks on the polychaete *Eulalia* sp., the surface layer of the proboscis epithelium partially peeled off (Figure 2C) and remained attached to the prey by sticky mucus secreted from the proboscis (Figure 3C–E). This layer contained numerous extruded pseudocnidae, embedded within the mucus. Unlike the Malykin et al. [18] scheme, the pseudocnidae do not remain on the proboscis surface but are released with the mucus, continuing to affect the prey even after the nemertean retracts its proboscis.

Feeding experiments with *C*. cf. *simula* show that contact with the proboscis rapidly immobilizes the prey within 5–45 s [18,55]. The limited number of putative toxic peptides and proteins detected in the proboscis proteome may reflect that prey immobilization is primarily mediated by TTX. However, the presence of putative ion channel-blocking toxins, like U-scoloptoxin [56] and MU-AGATOXIN-I [57], suggests that *C*. cf. *simula* can use a combination of neurotoxins that act synergistically with TTX. Such interactions could enhance the overall efficacy of the attack, leading to rapid and complete disruption of neuromuscular function. Additionally, putative protease inhibitors [58,59] also found in the proboscis secretions may suppress prey defense mechanisms—such as coagulation or proteolytic degradation—thereby facilitating toxin diffusion.

The present study provides the first proteomic analysis of the mucus of palaeonemerteans. Previous works have characterized mucus proteomes in several hoplonemerteans and two pilidiophorans [19,36,38,40]. In the mucus of *C*. cf. *simula*, we identified a putative pore-forming toxin, DELTA-alicitoxin-Pse2a, and two enzymes—acidic phospholipase A2 and sphingomyelinase C. DELTA-alicitoxin, originally described from a sea anemone [60], has been detected in *Cephalothrix* transcriptomes [34,37] but not in other nemerteans. Acidic phospholipases, widely distributed toxic enzymes in many animal venoms, have been reported in nemertean transcriptomes across all classes, but have not yet been confirmed at the protein level [37,40]. Sphingomyelinases, in contrast, have not been reported in nemerteans previously. We also identified a CRP with strong similarity to an ion channel-blocking neurotoxin, which was expressed at over 600-fold higher levels in the integument than in the proboscis, as well as two antimicrobial peptides. Structural comparison revealed conservation of loop orientation between the predicted neurotoxic peptide and Neurotoxin I, supporting a shared mode of interaction with ion channels. In contrast, variations outside the conserved disulfide-stabilized core may contribute to activity modulation or confer specificity toward nemertean targets, underscoring the functional diversity of mucus-associated CRPs. As in the case of the proboscis-expressed peptide, homologous sequences of the mucus CRPs were detected only in the transcriptome of *C*. *hongkongiensis*. In addition to proteinaceous toxins, the mucus of *C*. cf. *simula* contains relatively high concentrations of TTX, sufficient to repel or kill potential predators [53,61]. The coexistence of TTX and protein toxins likely represents a synergistic, multi-layered chemical defense, with TTX providing rapid predator deterrence and the proteins facilitating TTX penetration and contributing to longer-term protection and antimicrobial activity.

The CRPs identified in *C*. cf. *simula* likely represent novel toxin families in nemerteans. None of the selected peptides had matches in UniProt, and homologs were found only in the closely related species *C*. *hongkongiensis*, indicating a lineage-restricted distribution. Although certain structural features resemble those of conotoxins, the primary sequences are distinct, indicating unique molecular characteristics and potential functional innovations in nemertean peptide toxins.

## 4. Materials and Methods

### 4.1. Animal Collection

**Figure 4:**
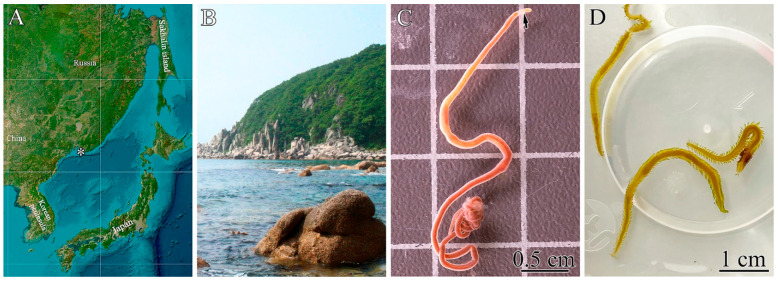
Type of locality and living specimens of *Cephalothrix* cf. *simula* and *Eylalia* sp. (**A**)—Geographical location of the sampling area (asterisk). (**B**)—Habitat of *C*. cf. *simula* and *Eylalia* sp. (**C**)—Live specimen of *C*. cf. *simula* (arrow indicates the head). (**D**)—Live specimens of *Eylalia* sp.

Specimens of *Cephalothrix* cf. *simula* and *Eylalia* sp. were collected during the summer seasons of 2022 and 2025 from rhizoids of the brown algae *Saccharina* sp. at a depth of 0.5–1.5 m in Spokoynaya Bay (42.7090 N, 133.1809 E), Sea of Japan (Figure 4A,B). The algal rhizoids were transported to the Vostok Marine Biological Station, A.V. Zhirmunsky National Scientific Center of Marine Biology, Far Eastern Branch of the Russian Academy of Sciences (Vladivostok, Russia), and maintained in tanks with aerated seawater at 17–20 °C until the nemerteans and polychaetes emerged. Collected animals were kept individually in aerated aquaria containing seawater sterilized through a 0.45 µm MF-Millipore™ membrane filter (Merck Millipore, Burlington, MA, USA) at 17 °C without feeding. Nemertean specimens were identified based on their morphology (Figure 4C) and partial sequences of mitochondrial cytochrome c oxidase subunit I (COI) gene according to Kuznetsov et al. [40]. Polychaetes were identified based on their morphology by Inna L. Alalykina, an expert in invertebrate zoology (Figure 4D).

### 4.2. Sample Preparation

Mucus samples from six *C*. cf. *simula* specimens were collected and processed as described in Kuznetsov et al. [40]. Briefly, individual specimens were placed in Petri dishes with 1 mL of sterile seawater, and mucus secretion was induced by a short electric pulse (6 V, 2 s) using copper electrodes. Four proboscis samples were obtained by relaxing individual specimens in 7% MgCl_2_ solution. Upon relaxation, the proboscis was fully everted, carefully extracted with forceps, and excised with a blade. All samples were then ultrasonicated and centrifuged, and the supernatants were purified using Oasis^®^ MCX solid-phase extraction cartridges (Waters, Milford, MA, USA). The eluates were dried under vacuum and stored at −20 °C until use.

### 4.3. Protein Extraction

Protein extraction was performed as described in Kuznetsov et al. [40]. Briefly, dry samples were dissolved in 8 M urea, reduced with 10 mM dithiothreitol, and alkylated with 20 mM iodoacetamide. Proteins were digested overnight with trypsin (1:50, *w*/*w*) at 37 °C, acidified with formic acid, and desalted using Strata-X solid-phase extraction cartridges (Phenomenex, Torrance, CA, USA). Eluates were dried under vacuum, resuspended in 50 μL of 1% aqueous formic acid, and filtered through 0.22 μm PVDF filters (Sigma-Aldrich, St. Louis, MO, USA).

### 4.4. HPLC-MALDI-TOF-TOF-MS/MS Analysis

RP-HPLC was performed on a NanoLC-Ultra 2Dplus system (SCIEX, Framingham, MA, USA) using a 0.1 × 100 mm Chromolith CapRod RP-18e HR column (Merck Millipore, Burlington, MA, USA) at 400 nL/min with 0.2% aqueous TFA (A) and 80% aqueous acetonitrile (B) at 22–24 °C. The column effluent was mixed with α-cyano-4-hydroxycinnamic acid matrix and calibration standards, fractionated with a micro-fraction collector (1 mm spots every 5 s, 1408 fractions per run), and analyzed by a TOF/TOF 5800 system (SCIEX, Framingham, MA, USA) in positive ion reflector mode. MS spectra were acquired at 2400 laser intensity, and MS/MS spectra at 3300 laser intensity with up to 20 top precursors (S/N > 40, 750–4000 Da) per spot.

### 4.5. Protein Identification and Bioinformatics Analysis

Mass spectra were processed with ProteinPilot™ v5.0 (SCIEX) using the Paragon 5.0.1.0 algorithm in thorough mode with biological modifications and substitutions enabled; carbamidomethylation of cysteine was set as a fixed modification. Spectra were searched against translated ORFs from the *C*. cf. *simula* transcriptome [37] plus common contaminants. Protein identifications with ≥95% confidence and detected in at least two samples were included in the final list. Proteomic data are available in Supplementary Files S1 and S2.

Annotation of protein sequences was performed using BLASTp (https://blast.ncbi.nlm.nih.gov/Blast.cgi, accessed on 15 April 2025) against the UniProtKB/Swiss-Prot database [48] and a database built from transcriptomic data of 12 nemertean species as reported by Vlasenko et al. [37] with the following parameters: maximum target sequences = 1, expect threshold (E-value) = 1 × 10^−5^, and substitution matrix = BLOSUM62. Sequence alignments and conservation analyses of CRPs were carried out in Jalview v2.11.5.1, using BLOSUM62 scoring matrices [49]. Signal peptides were predicted using SignalP v.6.0 [62]. Mature peptide regions were identified with the ConoPrec tool implemented in ConoServer [63]. Three-dimensional structures of CRPs were modeled and compared with known structures in the PDB using Phyre2.2 [50]. Structural alignments with the closest PDB templates were performed using the MatchMaker algorithm in UCSF Chimera v1.19 [51], which was also used for molecular surface and electrostatic potential analyses. Functional annotation of CRPs was performed using CSPred v. 1.1 [52].

### 4.6. qRT-PCR

The relative expression of five putative CRP toxins identified in the mucus and proboscis of *C*. cf. *simula* was evaluated by qRT-PCR. Five specimens were relaxed in 7% MgCl_2_, dissected, and proboscis and integument fragments were collected in RNAlater (Thermo Fisher Scientific, Waltham, MA, USA). Fragments were pooled by tissue type to generate two biological samples. Total RNA was extracted using TRIzol reagent (Thermo Fisher Scientific) according to the manufacturer’s instructions. RNA quality and quantity were assessed with a BioSpec-nano spectrophotometer (Shimadzu, Kyoto, Japan). Reverse transcription was performed on 2 µg of total RNA treated with RNase-free DNase I (New England Biolabs, Ipswich, MA, USA) using the MMLV RT kit (Eurogen, Moscow, Russia) in the presence of 1 U/µL RNase inhibitor (New England Biolabs, Ipswich, MA, USA). Reactions were incubated at 42 °C for 60 min and 70 °C for 10 min, then stored at −80 °C. Glyceraldehyde-3-phosphate dehydrogenase (*GAPDH*) and Actin-1 (*act-1a*) genes were used as reference genes. Primers for reference and CRP toxin genes were designed using the Primer-BLAST tool v3 [64] based on transcriptomic and proteomic data (Table 4). Primers were synthesized by Eurogen (Moscow, Russia), with the actin-1 gene primer obtained from Kuznetsov et al. [40]. RT-qPCR was performed using 5X qPCRmix-HS SYBR Master Mix (Eurogen) on a CFX96 Real-Time PCR system (Bio-Rad, Hercules, CA, USA). The program consisted of an initial denaturation at 95 °C for 2 min, followed by 42 cycles of 95 °C, 54 °C, and 72 °C for 15 s each, with a melting curve from 65–95 °C. Biological samples were analyzed in technical triplicate. Relative expression was calculated using the 2^−ΔΔCt^ method [65] with *act-1a* as reference and calibrated to proboscis levels.

### 4.7. Feeding Experiments

In the feeding experiment, three individuals of *C*. cf. *simula* and five individuals of *Eylalia* sp. were used. The nemerteans were placed in separate Petri dishes containing seawater at 17 °C, and after 30 min the polychaetes were introduced. Observations were recorded using a Canon EOS 6D Mark II camera (Canon Inc., Tokyo, Japan).

For TEM, both intact and experimental live worms were anesthetized in a 7% MgCl_2_ solution. The proboscis of the nemerteans was then dissected and cut into small fragments. Whole polychaete bodies were sectioned into fragments suitable for fixation. All fragments were fixed in 2.5% glutaraldehyde in 0.2 M cacodylate buffer (EMS, Hatfield, PA, USA) containing 0.15 M sodium chloride at room temperature, followed by post-fixation in 1% osmium tetroxide in distilled water for 1 h. The material was dehydrated through a graded ethanol and acetone series, embedded in Epon-Araldite resin (EMS, Hatfield, PA, USA), and sectioned into ultrathin slices (60 nm) using an Ultracut E ultramicrotome (Leica Biosystems, Wetzlar, Germany). The sections were stained with 1% uranyl acetate and 0.35% lead citrate, and examined with a Libra 120 transmission electron microscope (Zeiss, Jena, Germany).

## Figures and Tables

**Table 1:** Annotated toxin candidates identified in the proboscis proteome of *Cephalotrhrix* cf. *simula*.

Protein	UniProt Accession	Transcript ID	E-Value	Whole Seq. Length	Mature Peptide Length	Seq. Coverage (%)
Protease inhibitors						
Antistasin	P38977	ORF|026229	6.00 × 10^−20^	132	106	97
Leukocyte elastase inhibitor	Q5I0S8	ORF|039238	1.00 × 10^−103^	388	-	94
Neurotoxin						
U-scoloptoxin(05)-Er3a	P0DPY0	ORF|093517	1.00 × 10^−8^	144	121	92

**Table 2:** Annotated toxin candidates identified in the mucus proteome of *Cephalotrhrix* cf. *simula*.

Protein	UniProt Accession	Transcript ID	E-Value	Whole Seq. Length	Mature Peptide Length	Seq. Coverage (%)
Pore-forming toxin						
DELTA-alicitoxin-Pse2a	P58911	ORF|010939	3.00 × 10^−58^	424	404	86
		ORF|012501	5.00 × 10^−59^	445	425	92
		ORF|050110	8.00 × 10^−26^	236	216	83
Enzymes						
Acidic phospholipase A2 2	Q9W7J3	ORF|004160	4.00 × 10^−18^	160	138	69
Sphingomyelinase C	P17627	ORF|062113	2.00 × 10^−18^	356	317	72

**Table 3:** Functional classification of peptides identified in the proteome of *Cephalotrhrix* cf. *simula* according to CSPred v. 1.1.

Transcript ID	Probability Score				
	Ion Channel Blocker	Antimicrobial Peptide	Acetylcholine Receptor Inhibitor	Serine Protease Inhibitor	Hemolytic Peptide
Mucus					
ORF|114503	0.71	0.35	0.0	0.0	0.06
ORF|035733	0.03	1.0	0.03	0.0	0.2
ORF|002160	0.09	0.86	0.0	0.0	0.0
ORF|001398	0.13	0.05	0.0	0.0	0.29
Proboscis					
ORF|020829	0.13	0.19	0.0	0.0	0.01

**Table 4:** Primer sequences used for RT-qPCR and reaction efficiency.

Gene	Forward Primer 5′-3′	Reverse Primer 5′-3′	Amplicon
*C.sim_GAPDH*	TAATGACAACTGTACACGCA	TCGAAGCTGGGATAATGTTT	111 bp
*Uni_act-1a* ^1^	TCATCAGGGTGTCATGGT	AGGATACCTCTCTTGCTCTG	78 bp
*C.sim_ORF114503*	ATTTCTGGTGATTGATGGAGGG	TGGACATTCTCCATAAGTTGCT	136 bp
*C.sim_ORF035733*	CATGGCCTTGCATGGTTTAC	GTGACCGCGTTACCCATTT	113 bp
*C.sim_ORF002160*	GGCACTTCTTTTTCTGGTACAC	TACTGGCACCCCACCATTT	96 bp
*C.sim_ORF001398*	GCGTGTTTGTTTTTGCATCTC	CTTCGTCCCACTTCTCACC	107 bp
*C.sim_ORF020829*	AAAAACGGGGCTGTAATGGT	GTGACCTTCTTGGACACACA	134 bp

^1^ The primer was taken from Kuznetsov et al. [40].

## Data Availability

The original contributions of this study are included in the article and Supplementary Materials. Further inquiries can be directed to the corresponding author.

## Supplementary Materials

The following supporting information can be downloaded at: https://www.mdpi.com/article/10.3390/toxins18010017/s1. Table S1: Putative toxins in the proboscis proteome of *Cephalothrix* cf. *simula*; Table S2: Putative toxins in the mucus proteome of *Cephalothrix* cf. *simula*; Figure S1: Sequence alignments of cysteine-rich peptides from *Cephalothrix* cf. *simula* and *Cephalothrix hongkongiensis*, showing amino acid conservation, consensus sequences, and cysteine patterns; Figure S2: Structural comparison of predicted cysteine-rich peptides (CRPs) with their closest Protein Data Bank templates. (A) ORF|020829 (orange) superimposed on MU-AGATOXIN-I (gray). (B) ORF|114503 (orange) superimposed on NEUROTOXIN I (gray). Conserved cysteine residues are shown as yellow sticks; Figure S3: Molecular surface representation of the predicted antimicrobial peptide ORF|35733. (A) Electrostatic surface potential, with blue indicating positive charge and red indicating negative charge. (B) Hydrophobicity mapping, with blue representing the most hydrophilic regions, white intermediate, and orange-red the most hydrophobic patches; File S1: Proteomic data of the proboscis of *Cephalothrix* cf. *simula*; File S2: Proteomic data of the mucus of *Cephalothrix* cf. *simula*.
