# Supplementary material for: Proteinaceous Toxins in the Mucus and Proboscis of the Ribbon Worm Cephalothrix cf. simula (Palaeonemertea: Nemertea)
Source: Toxins (Basel). 2025 Dec 27;18(1):17. doi: 10.3390/toxins18010017 (PMC12846013; doi:10.3390/toxins18010017)
Supplement: Supplementary file 1 [file toxins-18-00017-s001.zip › Supplementary Figure S1-ms.pdf]

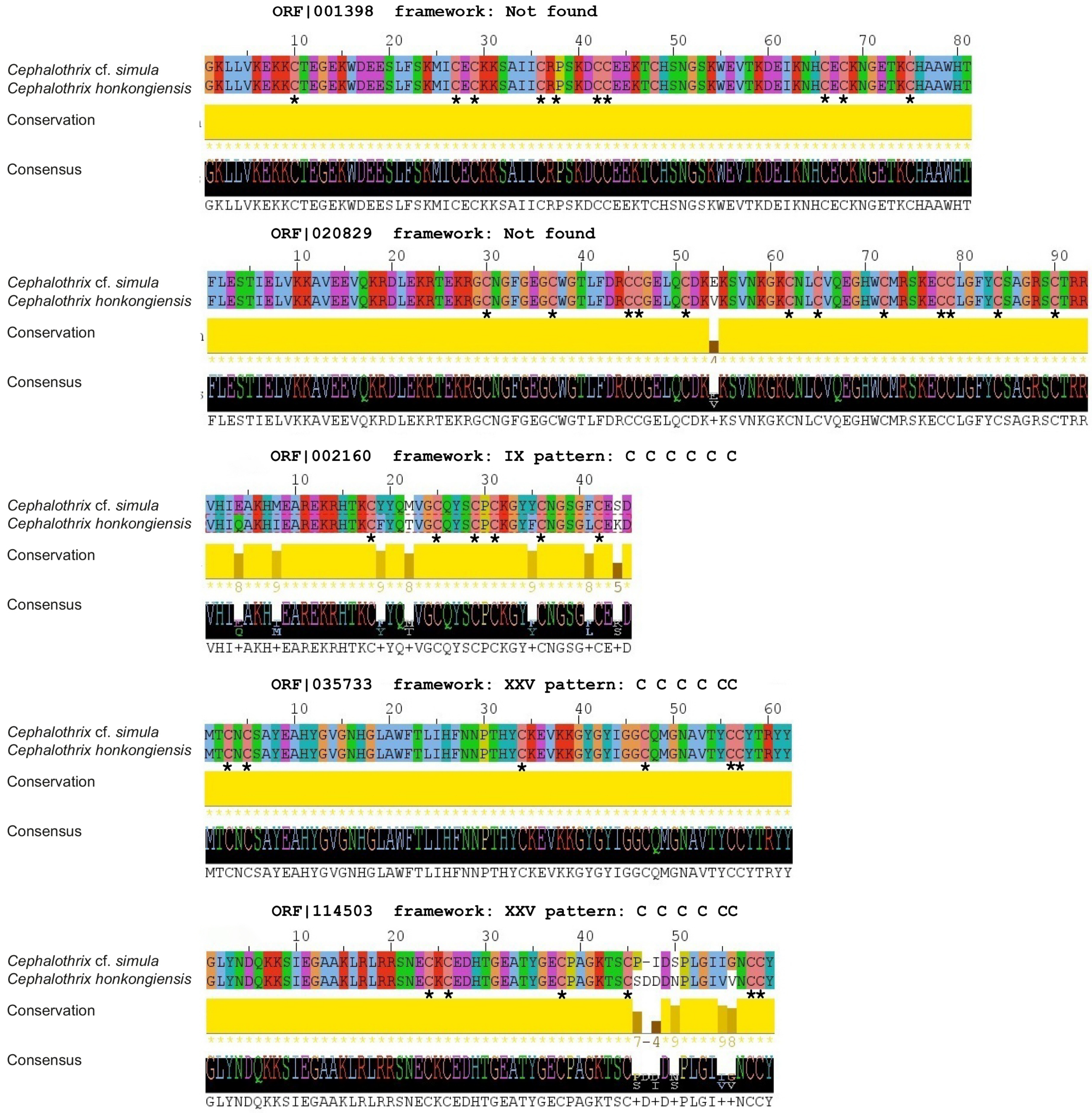
ORF|001398 framework: Not found
Cephalothrix cf. simula
Cephalothrix honkongiensis
GKLLVKEKKCTEGEKWDEESLFSKMICECKKSAIICRPSKDCCEEKTCHSNGSKWEVTKDEIKNHCECKNGETKCHAAWHT
GKLLVKEKKCTEGEKWDEESLFSKMICECKKSAIICRPSKDCCEEKTCHSNGSKWEVTKDEIKNHCECKNGETKCHAAWHT
Conservation
Consensus
GKLLVKEKKCTEGEKWDEESLFSKMICECKKSAIICRPSKDCCEEKTCHSNGSKWEVTKDEIKNHCECKNGETKCHAAWHT
ORF|020829 framework: Not found
FLESTIELVKKAVEEVQKRDLEKRTEKRGCNGFGEGCWGTLFDRCCGELQCDKEKSVNKGKCNLCVQEGHWCMRSKECCLGFYCSAGRSCTRR
FLESTIELVKKAVEEVQKRDLEKRTEKRGCNGFGEGCWGTLFDRCCGELQCDKVKSVNKGKCNLCVQEGHWCMRSKECCLGFYCSAGRSCTRR
FLESTIELVKKAVEEVQKRDLEKRTEKRGCNGFGEGCWGTLFDRCCGELQCDK+KSVNKGKCNLCVQEGHWCMRSKECCLGFYCSAGRSCTRR
ORF|002160 framework: IX pattern: C C C C C C
VHIEAKHMEAREKRHTKCYYQMVGCQYSCPCKGYYCNGSGFCESD
VHIQAKHIEAREKRHTKCFYQTVGCQYSCPCKGYFCNGSGLCEKD
VHI+AKH+EAREKRHTKC+YQ+VGCQYSCPCKGY+CNGSG+CE+D
ORF|035733 framework: XXV pattern: C C C C CC
MTCNCSAYEAHYGVGNHGLAWFTLIHFNNPTHYCKEVKKGYGYIGGCQMGNAVTYCCYTRYY
MTCNCSAYEAHYGVGNHGLAWFTLIHFNNPTHYCKEVKKGYGYIGGCQMGNAVTYCCYTRYY
MTCNCSAYEAHYGVGNHGLAWFTLIHFNNPTHYCKEVKKGYGYIGGCQMGNAVTYCCYTRYY
ORF|114503 framework: XXV pattern: C C C C CC
GLYNDQKKSIEGAAKLRLRRSNECKCEDHTGEATYGECPAGKTSCP-IDSPLGIIGNCCY
GLYNDQKKSIEGAAKLRLRRSNECKCEDHTGEATYGECPAGKTSCSDDDNPLGIVVNCCY
GLYNDQKKSIEGAAKLRLRRSNECKCEDHTGEATYGECPAGKTSC+D+D+PLGI++NCCY
