# Supplementary figures and images for: Proteinaceous Toxins in the Mucus and Proboscis of the Ribbon Worm Cephalothrix cf. simula (Palaeonemertea: Nemertea)
Source: Toxins (Basel). 2025 Dec 27;18(1):17. doi: 10.3390/toxins18010017 (PMC12846013; doi:10.3390/toxins18010017)

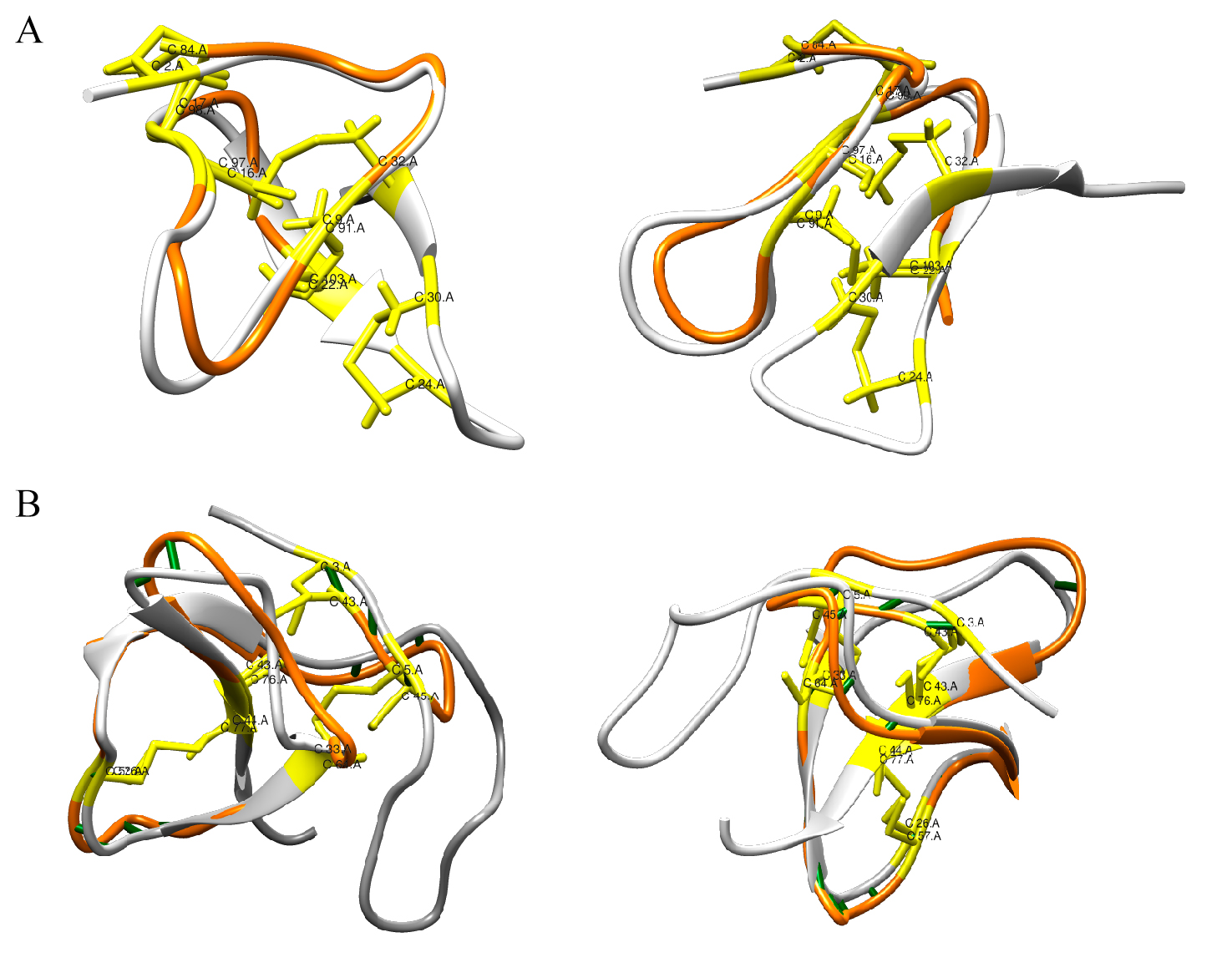

Supplement: Supplementary file 1 [file toxins-18-00017-s001.zip › Supplementary Figure S2.jpg]

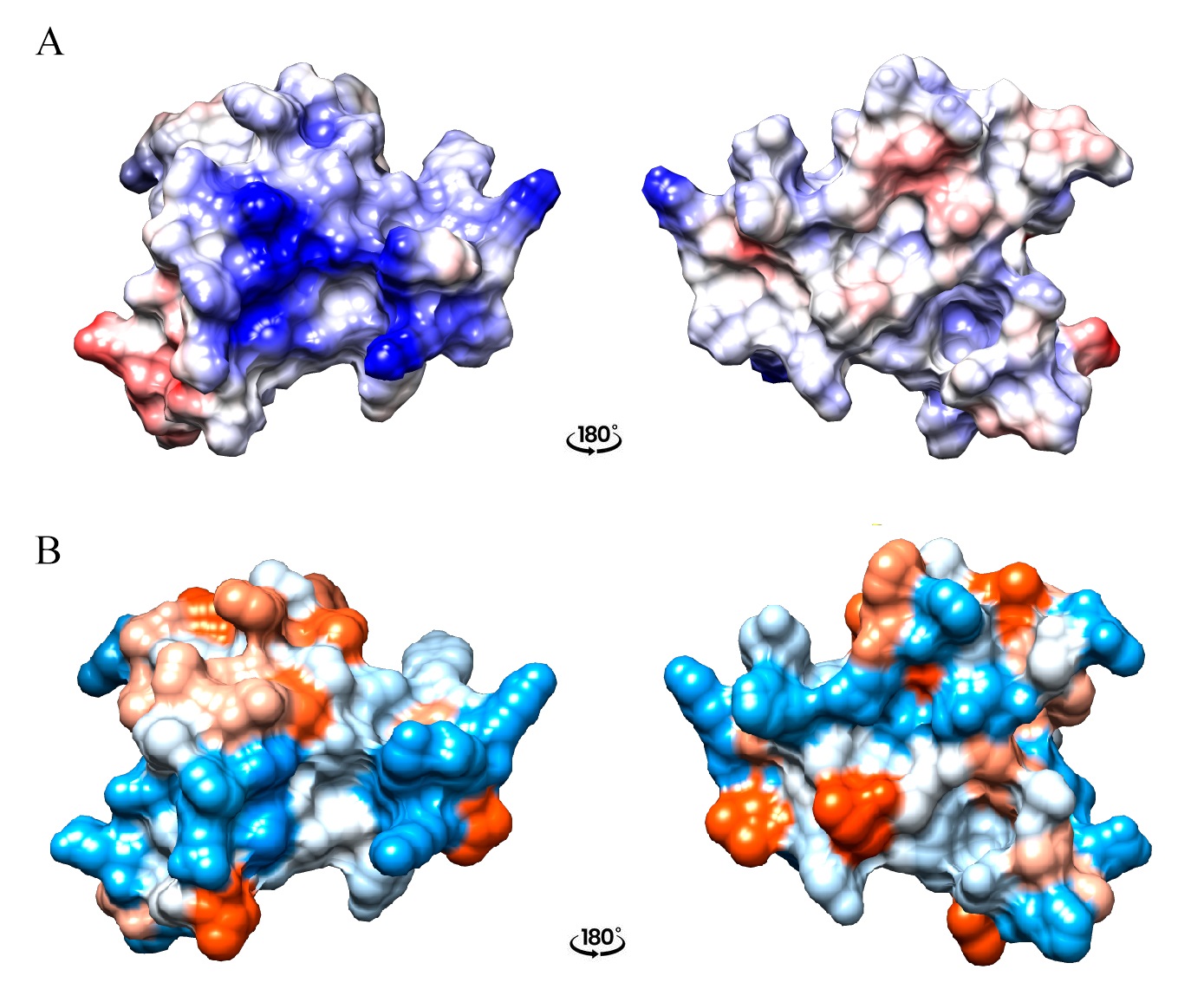

Supplement: Supplementary file 1 [file toxins-18-00017-s001.zip › Supplementary Figure S3.jpg]
